# Hemostasis at the edge between physiology and cancer

**DOI:** 10.1007/s11739-026-04294-1

**Published:** 2026-05-05

**Authors:** Angela Galardi, Elisa Dell’Orto, Francesca Bianchi, Giulia Cosentino, Valentina Fogazzi, Serenella M. Pupa, Marilena V. Iorio

**Affiliations:** 1 Microenvironment and Biomarkers of Solid Tumors Unit, Department of Experimental Oncology, Fondazione IRCCS Istituto Nazionale Dei Tumori Di Milano, Milan, Italy; 2 Department of Biomedical Science for Health, University of the Study of Milan, Milan, Italy; 3 Laboratorio Morfologia Umana Applicata, IRCCS Policlinico San Donato, Milan, Italy

**Keywords:** Hemostasis, Cancer, Coagulation, Platelets, Cancer-associated thrombosis, Circulating tumor cells, Tumor microenvironment, Immunothrombosis

## Abstract

Hemostasis lies at the edge between physiology and cancer, where the coagulation cascade—initiated by tissue factor, thrombin generation, and fibrin deposition—shifts from vascular repair to tumor promotion. The interplay between coagulation and cancer is now recognized as a two-way street, with tumor cells activating the clotting system and coagulation components feeding back to promote malignancy. Tumor cells not only foster proliferation and invasion by overexpressing tissue factor and activating Protease-Activated Receptors (PAR1/PAR2) signalling, but they also induce endothelial cells and fibroblasts in the tumor microenvironment (TME) to produce coagulation factors (TF, FV, prothrombin) through cytokines (VEGF, IL-1β), NF-κB signalling, and hypoxia factor (HIF-1α). Generated thrombin and FXa drive MAPK/PI3K pathways, angiogenesis (VEGF upregulation), and immune evasion by suppressing T-cell chemokines (CXCL9/10/11) and fostering M2 macrophages. Platelets, activated by tumor-associated coagulation, release PDGF, TGF-β, and VEGF to promote stromal remodeling and exclude cytotoxic T cells via PD-L1 transfer, while fibrin matrices shield tumors and recruit suppressive myeloid cells. This bidirectional interplay creates a protumoral niche supporting both primary growth and metastatic dissemination, where circulating tumor cells (CTCs) exploit platelet cloaking against shear stress and NK cells. Cancer hypercoagulability is a known state which elevates venous thromboembolism, with D-dimer as a prognostic biomarker linking thrombosis to aggressive disease. Targeting this hemostasis–cancer axis—via TF inhibition, anticoagulation, or antiplatelet therapy—offers therapeutic promise to disrupt proliferation, immune escape, and metastasis.

## Introduction

Hemostasis is the set of physiological mechanisms that allow the body to keep blood flowing within the vessels and, at the same time, to quickly stop bleeding in the event of vascular injury. When a thrombus forms, several molecular pathways of platelet activation and coagulation operate simultaneously in hemostasis and thrombosis [1]. The formation of a thrombus or platelet–fibrin clot is a highly controlled process that varies depending on the pathological context. In light of this, it is worth considering not only the biochemical pathways involved in thrombin generation and platelet activation, but also how these biochemical processes are influenced by other factors, including the contributions of endothelial cells, other blood cells, and local hemodynamic conditions [2]. The two systems are ancestrally related and represent defense mechanisms that limit infection by pathogens and stop bleeding at the site of vascular injury; it is therefore a context-dependent system [3]. On one hand, there are complement factors activating hemostasis, and on the other, there are coagulation proteins able to modulate complement. Complement and coagulation regulatory proteins strongly interact to modulate endothelial, platelet, and leukocyte function and phenotype**,** creating a potentially devastating amplification system that must be tightly regulated to avoid undesirable and collateral events as excessive bleeding or vascular occlusion and tissue ischemia [2, 4]. However, this finely balanced system becomes dangerous when the inflammatory response fails to resolve, as in chronic inflammation. In this context, persistent activation of the endothelium, platelets, and hemostatic pathways leads to a state of hypercoagulability and pathological immunothrombosis. Platelets are the main actors in thrombus formation and thrombin activation, and their activity can lead to serious consequences such as stroke and heart attack [5]. Tumors are also characterized by this imbalance, presenting an increased risk of thrombotic events, particularly venous thromboembolism (VTE). Various coagulation pathways, initially responsible for protecting the body, are in fact involved in tumor growth, invasion, and metastatic spread of malignant cells, as well as tumor-induced neoangiogenesis [6]. In this way, a physiologically protective system becomes maladaptive, transforming coagulation into an ally of tumor progression. The interplay between coagulation and cancer represents a complex bidirectional interaction; this form of the hypercoagulable state is associated with poor disease outcomes. Tumors promote the expression and activation of coagulation factors, which in turn drive proliferation, invasion, and immune evasion. Reciprocal interactions with tumor-educated platelets (TEPs) further support both local tumor growth and dissemination by shielding circulating tumor cells (CTCs) and priming the pre-metastatic niche [7]. Additionally, this hypercoagulable state appears to be a serious comorbidity and complication in cancer patients, often aggravated as a dangerous side effect of several anticancer therapies. Collectively, these findings position the coagulation system as a critical determinant of cancer progression, highlighting antiplatelet agents and anticoagulants as promising adjuvant therapies. In this review, we describe the most relevant molecular mechanisms underlying the bidirectional crosstalk between coagulation and cancer, from local hemostatic reprogramming within the primary tumor microenvironment to systemic immunothrombosis and metastatic dissemination. In addition, we discuss the clinical relevance of tumor-associated hypercoagulability, including thrombotic complications and biomarkers, and highlight emerging challenges in translating these insights into effective therapeutic strategies.

## Overview of the coagulation system

Haemostasis is characterized by two phases: the primary hemostasis, the initial process involving the formation of a temporary platelet plug at the site of injury; and the secondary hemostasis, which involves the coagulation cascade, where a series of clotting factors activated in a specific sequence stabilizes the platelet plug by forming fibrin strands that weave through the platelet aggregate, creating a more stable clot. The coagulation cascade is indeed a set of biochemical reactions leading to blood clotting, which occurs in response to blood vessel injury, thus facilitating tissue repair. This process is important for preventing excessive bleeding and promoting healing after injuries [8, 9]. The coagulation process was initially classified into two different pathways, extrinsic and intrinsic, which were shown to converge into a final common pathway by independently activating factor (F) X into FXa (FXa). In this model, the extrinsic pathway is initiated by TF exposure and activation of FVII, while the intrinsic pathway involves sequential activation of FXII, XI, IX, and VIII. However, this definition is nowadays considered obsolete and, although this model is still useful for understanding laboratory tests such as prothrombin time (PT) and activated partial thromboplastin time (APTT), according to the currently recognized cell-based model, the coagulation process is divided into three steps: initiation, amplification, and propagation [10].

One of the primary initiators of the extrinsic coagulation pathway is TF [11], which is constitutively expressed by adventitial cells surrounding blood vessels in physiological conditions and is released after vascular endothelial injury. TF can bind calcium and FVIIa (requiring vitamin K for activation) to activate FX. The activated FXa then binds to the surface of the tissue factor-expressing cell and converts a small amount of prothrombin (factor II) to thrombin. This is insufficient to convert adequate amounts of fibrinogen to fibrin; however, it triggers the following amplification step, where thrombin further locally activates platelets and co-factors V and VIII, which localize on the nearby surface of activated platelets, and FXI that also locally binds to platelets. At this point, after co-factor activation and localization on the platelet surface, a propagation phase takes place, with the assembly of highly efficient enzymatic complexes which boost thrombin formation leading to clot formation. FIXa binds to FVIIIa on the platelet surface to form the intrinsic tenase complex, which efficiently converts FX to FXa. After binding to its co-factor, FVa, FXa forms the prothrombinase complex responsible for the effective conversion of prothrombin to thrombin. In addition, FXIa produced during amplification activates FIX, responsible for further enhancing the whole process. The thrombin generated during propagation then cleaves soluble fibrinogen into unsoluble fibrin monomers. Afterwards, thrombin activates FXIII, which stabilizes the fibrin and the clot. Physiologically, coagulation factors are produced mainly by the liver; indeed, hepatocytes primarily synthesize most plasma coagulation factors (I, II, V, VII, VIII, IX, X, XI, XIII, proteins C and S) as they express the necessary vitamin K-dependent carboxylase enzymes. Also, endothelial cells can produce factor VIII and express TF upon activation, while also generating FV, FX, and prothrombin (FII) for surface-bound thrombin generation. Fibroblasts contribute to FV expression but have limited vitamin K-dependent factors due to lower γ-glutamyl carboxylase (GGCX) expression in comparison to endothelial cells [12].

## Reprogramming of Hemostasis in the Primary Tumor Microenvironment

**Fig. 1:**
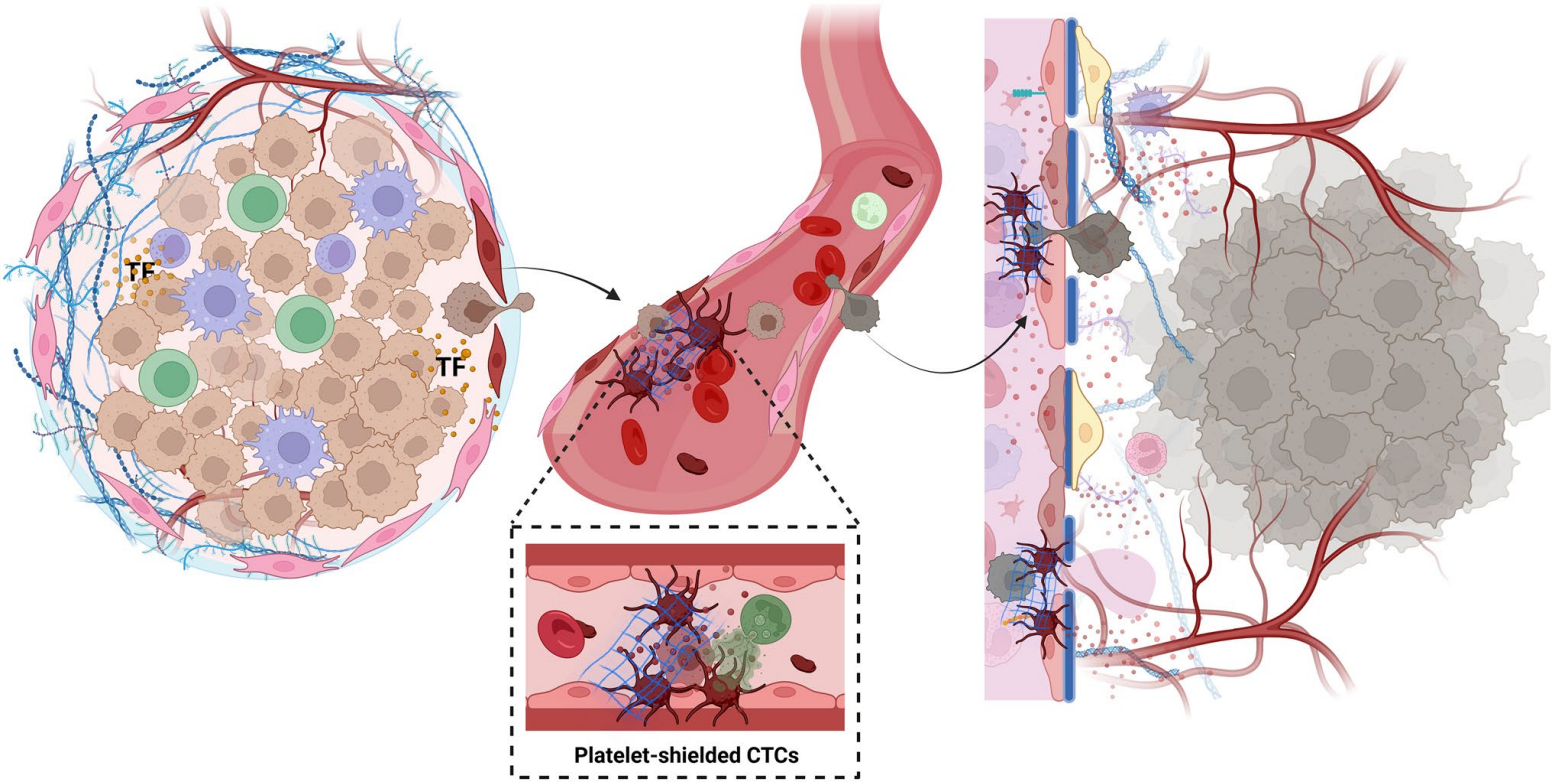
Hemostatic reprogramming from primary tumor to metastatic colonization. Tumor progression is accompanied by a progressive reprogramming of hemostatic pathway, from localized coagulation activation within the primary tumor microenvironment to systemic engagement and conditioning of distant tissues. Platelet- and coagulation-mediated support of circulating tumor cells promotes survival and vascular arrest, while persistent hemostatic activation contributes to the formation of a permissive pre-metastatic niche that facilitates metastatic colonization

The relationship between coagulation and cancer is now known as a two-way street, where tumor cells activate the coagulation system and its components to promote malignancy. Many cancers show a hypercoagulable state within the tumor microenvironment (TME), and coagulation factors and platelets can influence tumor initiation, progression, immune escape, and angiogenesis. Recent studies have shown that key coagulation elements, such as TF, thrombin, fibrin/fibrinogen, and platelets, influence the tumor microenvironment and tumor cell behavior through signalling-dependent crosstalk and paracrine interactions, thereby locally reprogramming hemostatic pathways to support neoplastic expansion (Fig. 1).

### Tissue factor–Factor VIIa–thrombin signalling and PAR1/PAR2 pathways

TF is a transmembrane protein that serves as the primary initiator of the extrinsic coagulation pathway [13]. TF is often overexpressed on the surface of tumor cells and vascular endothelial cells in different types of cancer, such as pancreatic cancer, acute lymphocytic leukemia, sarcomas, lung cancer, triple-negative breast cancer (TNBC), and glioma. In breast cancer, TF overexpression is associated with poor prognosis [14].

In addition to its usual role in hemostasis, TF functions as a signalling hub that actively shapes the TME. Specific protease-activated receptors (PARs) pathways are activated by the binding of circulating factor VIIa to TF as for PAR2 on tumor and stromal cells [15]. Through this signalling activity, TF exerts effects that extend beyond the tumor cell surface and propagate within the local microenvironment. The TF–FVIIa–PAR2 axis induces transcriptional programs associated with tumor progression, as activation of ERK/MAPK proliferation and PI3K survival pathways [16–18], upregulation of pro-angiogenic factors such as vascular endothelial growth factor (VEGF), inflammatory cytokines, and myeloid cell–recruiting chemokines [18]. Different studies show that TF knockdown halts progression in breast cancer models and compromises stemness potential. In this context, TF-positive extracellular vesicles (EVs) released by tumor cells act as local amplifiers of TF-dependent signalling, supporting the dissemination of procoagulant and pro-tumorigenic signals within the TME [19]. These EVs retain functional TF activity and promote tumor cell survival and extravasation, thereby reinforcing TF-driven paracrine communication, for instance, activating endothelial cells [20]. Studies in breast cancer models show that TF–PAR2 signalling suppresses the expression of T cell–recruiting chemokines such as CXCL9, CXCL10, and CXCL11, limiting CD8⁺ T cell infiltration and promoting immune exclusion within the primary tumor site [21]. Through these pathways, TF promotes neovascularization, stromal activation, and immune evasion within the primary TME [22]. In parallel, thrombin generated as a consequence of TF signalling activates PAR1, leading to further amplification of pro-inflammatory and pro-tumorigenic signals in neoplastic as well as stromal cells, such as endothelial cells and fibroblasts [23]. Thrombin, for instance, promotes angiogenesis through upregulation of VEGF and hypoxia-inducible factor (HIF-1α) [24], and induces tumor cell proliferation by triggering MAPK/PI3K pathways. Thrombin also increases the surface exposure of GPIIb–IIIa on platelets and tumor cells, thereby enhancing the interactions between tumor cells and platelets [25].

In pancreatic ductal adenocarcinoma, thrombin–PAR1 signalling skews the tumor microenvironment toward immunosuppression by fostering the accumulation of M2-polarized macrophages and excluding cytotoxic T lymphocytes, while simultaneously promoting desmoplastic stromal remodeling and angiogenesis [26]. These pathways together form a coagulation-driven axis that fuels tumor growth. The TF–PAR2 axis regulates cytokine and growth factor programs that modulate the tumor stroma. Thrombin–PAR1 supports pro-angiogenic, pro-fibrotic, and immune-evasive circuits. Notably, this axis promotes tumor growth in vivo by shaping the TMA to support neovascularization, immune escape, and invasive behavior [27]. Among coagulation factors that might contribute to tumor progression, it is also worth citing von Willebrand factor (vWF), which has emerged as a modulator of tumor–platelet interactions. vWF is a multimeric plasma glycoprotein, produced by endothelial cells and megakaryocytes [28]. vWF has a central role in primary hemostasis, where it mediates platelet adhesion at sites of vascular injury. Tumors can sequester circulating vWF into the tumor stroma [29], where it may facilitate cancer progression and metastasis through interactions mediated by its collagen-binding domains. This mechanism highlights how circulating hemostatic factors can be locally repurposed without systemic activation. It is reported that the main role in this process is played by the collagen–binding motif within the A3 domain of vWF [30]. How do tumors sustain and amplify local coagulation within the tumor microenvironment to foster their own growth? Tumors are known to corrupt the surrounding microenvironment to pro-tumoral and immune suppressive components, and the local induction and recruitment of coagulation factors represent a key component of this reprogramming process. Through cytokine production, activation of hypoxia-driven pathways, and direct cell–cell interactions in the TME, tumors induce tissue endothelial cells (TE) and other stromal cells to locally produce coagulation factors. For instance, neoplastic cells release VEGF, IL-1β, and TNF-α, which upregulate TF expression on endothelial cells via NF-κB activation [31], leading to spatially confined local FVIIa assembly and thrombin generation.

HIF-1α in the hypoxic TME further boosts TF synthesis in endothelial cells and fibroblasts, amplifying coagulation signalling within the primary tumor independently of tumor cell-derived TF [32]. Another component of the TME engages in a bidirectional crosstalk with the coagulation system: M2-polarized tumor–associated macrophages (TAMs) express TF [33]; on the other hand, coagulation factors contribute to their pro-tumoral differentiation [34]. These coagulation-driven signals also engage platelets, establishing a crucial tumor–platelet crosstalk that shapes the primary TME.

### Platelets in tumor growth and angiogenesis

Within the primary TME, platelets are activated as a direct result of tumor-driven, coagulation-dependent signaling. TF, via PARs, is one of the focal factors upstream of platelet activation, while direct interactions between tumors and platelets, as well as soluble tumor-derived agonists, promote continued activation [35]. Thrombin-mediated platelet activation via protease-activated receptors starts selective and context-dependent platelet degranulation, resulting in the release of bioactive mediators that influence tumor biology [36]. Platelet-derived growth factors include: platelet-derived growth factor (PDGF), VEGF, and transforming growth factor-β (TGF-β). These factors promote stromal activation, angiogenesis, and immune suppression within the primary tumor [35, 37]. PDGF and TGF-β stimulate fibroblast activation and extracellular matrix remodeling [38], while VEGF drives the angiogenic switch. In parallel, platelet-derived TGF-β impairs cytotoxic immune responses, contributing to local immune evasion within the tumor niche [39]. Moreover, continued tumor–platelet crosstalk leads to the emergence of tumor-educated platelets (TEPs), which exhibit altered secretory profiles that further reinforce tumor-supportive signaling. These locally conditioned platelets act as amplifiers of coagulation- and tumor-derived signals, extending platelet-mediated effects within the primary tumor site. Collectively, platelet activation in the context of locally reprogrammed coagulation establishes a permissive microenvironment that indirectly supports tumor growth by adding stromal remodeling, angiogenesis, and immune suppression.

### Fibrinogen and Fibrin as drivers of tumor-supportive matrix remodeling

Fibrinogen is the most common clotting factor that polymerizes into fibrin. It plays a crucial role in TME, in particular by involving locally activated coagulation in extracellular matrix remodeling, inflammation, and immune regulation. Activation of the coagulation cascade leads to the conversion of fibrinogen into fibrin, which is deposited in the tumor stroma, forming a transient but bioactive extracellular matrix [40]. Several studies have demonstrated that this fibrin system acts as a mechanical and signalling scaffold that promotes the adhesion, migration, and invasion of tumor cells, as well as supporting angiogenesis through the binding and local concentration of pro-angiogenic factors [40]. In addition, fibrin matrices provide an adhesive platform for platelets and leukocytes, reinforcing coagulation-dependent cellular interactions within the TME. In parallel, fibrin-rich extracellular matrices contribute to immune modulation by favoring the retention and functional polarization of TAMs and limiting effective cytotoxic T cell infiltration, thereby promoting immune exclusion within the primary tumor site [38]. Fibrin(ogen) accumulation downstream of locally reprogrammed coagulation establishes a mechanoactive, pro-angiogenic, and immunomodulatory matrix that indirectly sustains tumor progression.

The coagulation-determined remodeling of the TME not only provides structural and biochemical support for tumor growth but also changes the spatial and cellular architecture of the tissue. This reorganization influences the recruitment, localization, and functional polarization of immune cells.

### Coagulation–associated immune modulation in the tumor microenvironment

Hemostasis and immunity are strictly interconnected, and within the TME, coagulation factors and platelets influence immune cell behavior through multiple mechanisms. Platelets can transfer inhibitory signals via the immune checkpoint ligand PD-L1, thereby promoting tumor cell evasion from T cell–mediated surveillance [41]. Platelets also release transforming TGF-β1, which suppresses cytotoxic immune responses and limits effective anti-tumor immunity within the primary tumor site [42]. Another mechanism of immunomodulation mediated by coagulation involves fibrin and fibrinogen. Fibrinogen binds myeloid cells through the integrin αMβ2 (Mac-1), promoting the accumulation of myeloid populations within the TME. Although initially associated with inflammatory activation**,** these cells frequently shift toward immunosuppressive phenotypes in the tumor context [43]. TAMs accumulating in fibrin-rich areas commonly switch to an M2-like program that supports tissue remodeling and tumor progression while impairing anti-tumor immune surveillance [26]. Coagulation proteases further contribute to immune modulation by acting as signalling molecules on immune cells. Thrombin activates platelets and signals through PAR1 expressed on dendritic cells and T lymphocytes, and elevated thrombin activity has been associated with impaired dendritic cell maturation and reduced T cell proliferation within tumors. Similarly, TF–FVIIa signalling can induce tumor cells to secrete growth factors and cytokines that expand immunosuppressive cell populations, including myeloid-derived suppressor cells and M2-polarized macrophages [44]. These cells limit T cell activity through nutrient depletion, such as arginase-mediated arginine consumption, and by expressing inhibitory checkpoint ligands, thereby reinforcing a locally immunosuppressive loop within the TME [41]. These coagulation-driven mechanisms converge to suppress anti-tumor immunity within the primary TME, positioning immune modulation as a direct consequence of locally confined hemostatic reprogramming.

## From local dysregulation to systemic activation: immunothrombosis and cancer

Although coagulation-driven immune modulation is limited to the primary TME, persistent tumor-associated hemostatic activation can progressively overcome local regulatory barriers. As this loss of spatial and temporal control unfolds, coagulation and innate immune pathways converge into immunothrombosis, a host defense program that becomes chronically engaged in cancer. In this context, immunothrombosis is a critical transition point through which locally reprogrammed hemostasis expands systemically, shaping thrombotic risk, inflammation, and tumor dissemination (Fig. 1).

### Immunothrombosis as a physiological host response

Immunothrombosis is an evolutionarily conserved host-defense program in which innate immune cells, platelets, the endothelium, and the coagulation cascade cooperate to contain danger signals and preserve vascular integrity [45]. In physiological conditions, this response is spatially restricted and temporally self-limited: microvascular thrombi and fibrin-rich scaffolds can localize inflammatory cues, support leukocyte recruitment, and physically constrain pathogen or damage-associated dissemination [46]. Neutrophils are central effectors of this mechanism/system, not only through the release of cytokines and proteases, but also through neutrophil extracellular traps (NETs), which are capable of improved platelet adhesion and activation of coagulation factors [47, 48]. In parallel, platelet–leukocyte interactions coordinate thromboinflammatory signalling, integrating vascular surveillance with rapid hemostatic sealing. Immunothrombosis is conceptually distinct from “thromboinflammation” as pathology: it is best viewed as a thresholded, context-dependent program that becomes detrimental when continued or unresolved [46, 49].

### Chronic activation in cancer: loss of local restriction in cancer

Cancer is a continued source of tissue stress [50]. This is characterized by inflammation, hypoxia, and vascular disruption [51]. Chronic involvement of immunothrombosis leads to extravasation [52]. This activates and propagates haemostatic–immune circuits in the vascular stream, promoting a systemic prothrombotic and proinflammatory state. In this context, platelet activation, endothelial dysfunction, and coagulation protease signalling reinforce innate immune activation [53]. However, compromised resolution mechanisms favor sustained thrombin generation and fibrin turnover. This suggests a shift from immunothrombosis, a protective response by the host, to a chronically involved process linking local tumor biology with systemic thrombosis and inflammation [54].

### Neutrophils, NETs, and Platelet–Leukocyte Crosstalk

The transition from *“regulated to amplified”* immunothrombosis in cancer is regulated by neutrophils and their crosstalk with platelets [55]. In particular, the activated neutrophils release NETs—chromatin fibers decorated with granular proteins—which act as scaffolds that trap cells and concentrate procoagulant activity, thereby supporting activated platelet adhesion and amplification of coagulation factor activation [56, 57]. Beyond thrombosis, these aggregates can rewrite vascular–immune interfaces: NETs and platelet-derived mediators may contribute to endothelial activation, leukocyte recruitment patterns, and local immune suppression, thereby coupling thrombotic propensity to tumor-permissive inflammation [58]. In cancer, the clinical and experimental literature places NET-driven immunothrombosis at the intersection of thrombotic complications and metastatic efficiency, as NETs can capture circulating tumor cells and facilitate colonization in permissive niches and have been implicated in mechanisms of therapy resistance [59, 60].

## Coagulation in metastatic dissemination

Coagulation-related mechanisms involving platelets, fibrin, and innate immune cells help circulating tumor cells to survive in the bloodstream and spread to distant organs, while preparing permissive pre-metastatic niches. Therefore, coagulation actively contributes to metastatic spread and colonization beyond the primary tumor site [61] (Fig. 1).

### Platelet-mediated defense and immune evasion of circulating tumor cells

Platelets are among the earliest and most effective *“friends”* of CTCs during hematogenous dissemination [62], and they exert this role through different actions:

- They ensure physical protection by platelet aggregation, a phenomenon known as tumor cell-induced platelet aggregation (TCIPA), which leads to the formation of a coating that physically protects CTCs [15]. This coating evades direct recognition of tumor cells by natural killer (NK) cells and cytotoxic lymphocytes, thereby improving the survival of CTCs in circulation [63];

- Platelet–CTC interactions actively reprogram immune recognition. Platelet-derived transforming TGF-β suppresses NK cell cytotoxicity and downregulates activation receptors, while platelet transfer of major histocompatibility complex class I (MHC-I) molecules to tumor cells can cause a “pseudo-self” phenotype that further compromises immune clearance [64];

- Platelet activation promotes local thrombin generation and fibrin deposition around CTCs, stabilizing platelet–tumor aggregates and facilitating vascular arrest at distant sites [65];

Collectively, platelet shielding represents a critical hemostatic mechanism that combines immune evasion with enhanced survival and metastatic efficiency of CTCs.

### Coagulation–mediated vascular arrest and extravasation

After surviving in the circulatory system, CTCs must stop within the microvasculature and extravasate into distant tissues to establish metastatic lesions [66]. The coagulation factors play a key role in this phase by promoting the adhesion of tumor cells, platelets, fibrin, and the endothelium [67]. Local thrombin generation and fibrin deposition around circulating tumor cell–platelet aggregates enhance vascular arrest by stabilizing tumor cell adhesion to the vessel wall [68]. In parallel, platelet activation and coagulation proteases increase endothelial permeability and disrupt junctional integrity, thereby facilitating tumor cell transendothelial migration [69, 70]. Platelet-derived mediators, including transforming TGF-β and VEGF, promote endothelial reprogramming and vascular leakage at sites of arrest. Through these mechanisms, coagulation not only supports the physical trapping of tumor cells but also actively orchestrates the extravasation process that enables metastatic seeding at distant organs [71].

### hemostatic control of metastatic niche formation and outgrowth

Metastatic colonization is preceded by the formation of a pre-metastatic niche, in which tumor-derived and host-derived factors remodel distant tissues before the arrival of circulating tumor cells [72]. Increasing evidence suggests that coagulation components, including platelets, fibrin, and NETs, contribute to this early conditioning by promoting vascular activation, immune modulation, and stromal remodeling [73]. Beyond vascular arrest and extravasation, hemostatic mechanisms continue to shape the microenvironment of secondary organs, influencing metastatic niche formation and tumor outgrowth. Fibrin deposition and platelet accumulation at early metastatic sites create a provisional matrix that supports tumor cell survival, protects against immune-mediated clearance, and provides a scaffold for stromal and endothelial cell recruitment [59]. In addition, coagulation-driven inflammation and immune modulation contribute to the establishment of a tumor-permissive metastatic niche [59, 60]. Through these coordinated mechanisms, hemostasis exerts long-lasting control over the efficiency and stability of metastatic growth beyond the primary tumor site.

## Systemic consequences of tumor-driven coagulation

### Cancer-associated thrombosis as a maladaptive systemic response

It is now well estabished that the presence of a neoplasm causes a profound alteration in the body's homeostasis, resulting in a condition of systemic dysregulation characterized by the impairment of normal inter-systemic regulatory mechanisms. This condition manifests itself through persistent, amplified, and maladaptive biological responses, which involve the immune, inflammatory, coagulation, and endothelial systems in an integrated manner. The consequence is the establishment of a chronic inflammatory state and a marked systemic pro-thrombotic predisposition. The dysregulation observed therefore represents a pathological reorganization of the host’s biological networks, functionally oriented towards promoting the survival, progression, and metastatic spread of the neoplastic disease. The persistent activation of the inflammatory–coagulatory axis results in a condition of systemic hypercoagulability, mediated by specific molecular effectors expressed by tumor cells and activated endothelium, among which TF plays a predominant role. Elevated levels of TF in tumor tissue are associated with fibrin deposition, the development of thrombosis, and venous thromboembolism (VTE), contributing to an unfavorable clinical prognosis [54].

Cancer-associated VTE is one of the main clinics for prothrombotic states and has very serious consequences for cancer patients, who risk delaying or having to interrupt the systemic therapy they are undergoing. VTE rates depend on the type of tumor, with pancreatic, stomach, and primary brain tumors presenting the highest risk, as well as on individual factors relating to the patient and cancer treatment [74]. Tatsumi divided tumors into three groups based on their risk of VTE: high risk (pancreatic, ovarian, brain, gastric, gynaecological, and hematological), intermediate risk (colon and lung), and low risk (breast and prostate) [75]. Considering that VTE is the second leading cause of death in patients with cancer, it is essential to assess individual thrombotic risk before starting treatment. To this end, the Khorana score, developed in 2008, is commonly used. It takes into account the location of the tumor, platelet count, haemoglobin levels, white blood cell count, and body mass index. This score allows cancer patients undergoing chemotherapy to be stratified according to their risk of developing VTE. Based on the total score, patients are classified into low-risk (0.3–0.8%; score 0), intermediate-risk (1.8–2.0%; score 1–2), and high-risk (6.7–7.1%; score ≥ 3) groups. However, most studies evaluating the clinical utility of these stratification systems have shown that these scores are only applicable to certain types of cancer and are not universally applicable. This is therefore a limitation, and it may be necessary to develop specific risk models for each type of cancer, based on specific biomarkers or clinical and biological characteristics [75]. In this context, TF-positive microvesicles (MVs) released by tumor cells and cells in the micro-environment represent a potential mechanism for systemic amplification of the prothrombotic state. These MVs, by conveying functionally active TF into the circulation, contribute to the dissemination of procoagulant activity beyond the primary tumor site, promoting coagulation activation in distant vascular districts through activation of the extrinsic pathway with consequent production of fibrin clots in the vessels*. Geddings *et al. demonstrated that MVs released from a pancreatic adenocarcinoma cell line expressing high levels of TF (BxPc-3) trigger thrombin generation in the presence of plasma in vitro, identifying thrombin as the main indirect mediator of platelet activation. In vivo, they demonstrated that the injection of MVs derived from the BxPc-3 line into wild-type mice increased TF activity by approximately tenfold compared to mice with localized tumors. This resulted in increased platelet activation, fibrin deposition, and increased venous thrombosis, thus demonstrating a causal role of TF + MVs in cancer-associated thrombosis [76].

### Bidirectional crosstalk and biomarkers of thrombotic risk in different types of cancer

The activation of coagulation is not a unidirectional process, but is part of a complex bidirectional crosstalk between the tumor and the hemostatic system. The generation of thrombin and platelet activation contributes to promoting angiogenesis, invasiveness, and immune protection of tumor cells. It has been demonstrated that thrombin and fibrin are responsible for the immune escape of tumor cells as they help the formation of platelet aggregation around circulating tumor cells (CTCs), allowing the latter to escape the activity of Natural Killer (NK) cells [77]. TF associated with tumor cells not only plays a thrombogenic role but also exerts direct pro-tumor functions by promoting the adhesion of CTCs to the endothelium. Evidence of bidirectional crosstalk is demonstrated by the fact that certain biomarkers of the coagulation system, such as fibrinogen, D-dimer, thrombin–antithrombin complexes (TAT), fibrin degradation products (FDP), prothrombin time (PT), and activated partial thromboplastin time (APTT), reflect the state of hypercoagulability associated with cancer, tumor stage, and risk of metastasis. These biomarkers can be defined as readouts because, although they are observable and quantifiable molecules, they do not represent a primary causal indicator but reflect the functional state of activation of the inflammation–coagulation axis, resulting from a condition of systemic dysregulation induced by the presence of the tumor mass (Table 1). *Yi-Qing Shen *et al*.* have shown that all coagulation biomarkers are significantly associated with the progression of gastric cancer. In particular, APTT, PT, and TT show a significant correlation with increased TNM stage, indicating progressive involvement of the coagulation system in the more advanced stages of the disease. Conversely, fibrinogen degradation products (FDP) and D-dimers are significantly associated with the presence of distant metastases, suggesting intense activation of fibrinolytic processes in patients with disseminated disease. Overall, the alteration of these parameters reflects a pathological and systemic activation of the coagulation and fibrinolytic axes, closely related to the clinical development and progression of gastric cancer [78].

**Table 1:** Coagulation biomarkers reflecting tumor-associated hypercoagulability and clinical outcome

Biomarkers	Pathways	Clinical relevance in cancer
Fibrinogen	Coagulation cascade	Elevated plasma fibrinogen levels are associated with increased risk of metastasis in gallbladder and gastric cancer [79–81]
D-dimer	Fibrinolytic system	Elevated D-dimer levels are associated with poor prognosis in lung, pancreatic, metastatic gastrointestinal cancers [82–84]
Thrombin-Antithrombin Complexes (TAT)	Thrombin activation	Thrombin-antithrombin complex causes high risk of thrombosis and TEV in colorectal, gastric, cervical and non-small-cell lung cancer [85–87]
Fibrin Degradation Products (FDP)	Fibrinolysis	High FDP levels are associated with advanced TNM stage and poor prognosis in esophageal and laryngeal squamous cell cancer [88, 89]
Prothrombin Time (PT)	Coagulation pathway	Prolonged preoperative PT predicts postoperative recurrence in colorectal cancer patients [90] and gastric cancer patients with distant lymph node metastasis [91]
Activated Partial Thromboplastin time (APTT)	Coagulation pathway	Prolonged APTT is associated with shorter overall and progression-free survival in multiple myeloma [92]

## Thrombotic events as side effects of anti-cancer drugs

### Impact of anticancer therapies on the coagulation mechanism

Anticancer therapies have a significant and well-recognized impact on coagulation mechanisms. First, the most commonly used cytotoxic chemotherapies, although many have improved in terms of off-target toxicity, induce damage to the epithelium of blood vessels, the endothelium, through multiple and often converging mechanisms, including oxidative stress, mitochondrial damage, activation of *the* innate immune system and consequently of a strong inflammatory response, lately culminating in endothelial cell apoptosis [93–96]. Anthracyclines, cisplatin, and taxanes are among the agents with the greatest impact on the endothelium. The former agents generate reactive oxygen species and cause endothelial Nitric Oxide Synthase (eNOS) uncoupling, reducing nitric oxide bioavailability and promoting vascular dysfunction; cisplatin directly induces endothelial cell apoptosis*,* and taxanes also contribute to endothelial damage by interfering with the cytoskeleton and consequently with endothelial cell migration and proliferation [93, 97, 98]. Overall, these effects convert the endothelium from an antithrombotic surface to a procoagulant surface, predisposing to early and late thrombosis and vascular complications. Chemotherapy-induced endothelial damage reduces the expression and function of key anticoagulant molecules, such as thrombomodulin, protein C*,* and antithrombin, impairing normal control of thrombin generation. At the same time, endothelial apoptosis promotes the release into the circulation of endothelial particles rich in anionic phospholipids, with high procoagulant activity [99–102]. Microparticles are fragments released from stimulated or apoptotic cells after plasma membrane budding. In biological fluids, they represent reliable cell damage biomarkers. It has been shown that, particularly during cisplatin treatment, these microparticles sustain thrombin generation through phospholipid-dependent and largely tissue factor–independent mechanisms, representing an alternative pathway of coagulation amplification. Antiangiogenic treatments also alter coagulation mechanisms, resulting in prothrombotic effects. Anti-VEGF antibodies and tyrosine kinase inhibitors that block VEGF signaling by targeting the ligand or its receptors exert well-known prothrombotic effects [93, 103, 104]. VEGF inhibition leads to reduced nitric oxide production, increased vascular tone, endothelial dysfunction, and increased expression of pro-adhesive and procoagulant molecules. Clinically, these effects translate into an increased incidence of arterial and venous thrombotic events, hypertension, and systemic vascular damage. In addition to altering endothelial function and integrity, thus affecting the coagulation cascade downstream, anticancer therapies frequently induce quantitative and qualitative platelet alterations, affecting platelet production upstream. Thrombocytopenia is mainly caused by suppression of bone marrow hematopoietic activity, with both reduced megakaryopoiesis and increased apoptosis of platelet precursors [105]. Anthracyclines, platinum compounds*,* and taxanes may also interfere with platelet maturation signaling [93]. Beyond numerical reduction, chemotherapy promotes functional alterations of circulating platelets, with increased activation and adhesiveness in response to injured vascular surfaces and inflammatory signals. This contributes to an unstable hemostatic state, in which thrombocytopenia may coexist with increased thrombotic tendency, especially in the presence of a dysfunctional endothelium. In relation to these processes, anticancer therapies induce specific alterations in plasma coagulation factors, usually reported as markers to monitor thrombotic risk. During cisplatin-based chemotherapy regimens, a significant increase in vWF and FVIII levels has been documented [94, 106]. Both are produced or released by activated endothelium and are considered sensitive markers of endothelial damage*,* and their increase is associated with enhanced thrombin generation and a higher risk of early venous and arterial thromboembolic events. In parallel, alterations in the fibrinolytic system may occur, with increased Plasminogen Activator Inhibitor-1 (PAI-1) levels, mostly from cancer-associated fibroblasts (CAFs), and reduced fibrinolytic activity, further contributing to the persistence of a procoagulant state during and shortly after cancer treatment [94, 107].

### Role of pharmacological interactions between anti-cancer drugs and anticoagulants

Pharmacological interactions between anticancer therapies and anticoagulants are frequently mediated by interference with hepatic metabolism and cell membrane-transport systems, and consequently with plasma drug levels. Several direct oral anticoagulants (DOACs) are substrates of these systems depending on both cytochrome P450 (e.g. CYP3A4) and P-glycoprotein (P-gp) [108–110]. Therefore, in cancer patients, drugs acting as CYP3A4 and/or P-gp inhibitors or inducers can significantly alter DOAC effects. Modulation of CYP3A4 and P-gp pathways leads to changes in anticoagulant plasma levels, either increasing or reducing drug availability. In addition, reduced gastrointestinal absorption may also occur. Indeed, in cancer patients indirect pharmacodynamic interactions are common, such as chemotherapy-induced nausea, vomiting, or diarrhea, which can impair oral anticoagulant absorption [111, 112]. It is therefore evident that pharmacokinetic interactions during simultaneous or closely timed treatment with anticancer drugs may lead to reduced anticoagulant effect with increased thrombotic risk, or even conversely to increased anticoagulant plasma levels with higher bleeding risk [113]. This risk is particularly relevant in cancer patients, in whom predisposing factors such as thrombocytopenia, mucositis, or organ dysfunction frequently coexist [114, 115]. Tyrosine kinase inhibitors (TKIs) are themselves associated with myelotoxicity and bleeding, further increasing hemorrhagic risk when combined with DOACs or warfarin [112, 113]. For this reason, guidelines recommend systematic assessment of drug–drug interactions (DDIs) before starting or modifying anticoagulant therapy in cancer patients [115, 116]. The concomitant management of oncologic therapies and anticoagulation is complex and requires an individualized approach [95]. Therapeutic decisions must integrate thrombotic or bleeding risk, tumor characteristics, and drug interactions. In cardio-oncology, this approach has been formalized in decision-making frameworks that explicitly include DDIs as a core element of therapy selection. However, the lack of definitive clinical data for many drug–drug combinations often requires dynamic treatment adjustments, including dose changes or switching between anticoagulant classes (e.g., from DOACs to low-molecular-weight heparins), further increasing management complexity over time [114, 117].

### Anticoagulants in cancer immunotherapy

In addition to the most common regimens based on chemotherapy and molecular targeted drugs, in recent years the use of immunotherapy has grown, both for the treatment of advanced-stage tumors and before surgery of the primary tumor. Studies that have evaluated the effect of anticoagulant drugs on the efficacy of immunotherapy are largely retrospective, and the results are not consistent. Interactions between immunotherapy and anticoagulants are complex: preclinical data suggest a possible therapeutic synergy between immune checkpoint blockade and inhibition of the coagulation cascade. The hypothesis is that anticoagulants can enhance the antitumor efficacy of immunotherapy by improving tumor blood flow and therefore immune cell access, and by reducing tumor hypoxia and therefore tumor metabolic escape strategies [118, 119]. However, whereas some clinical studies show a benefit, others indicate no significant difference, along with an increased bleeding risk. To date, available evidence is not sufficient to support a prospective evaluation of the combination of anticoagulation and immunotherapy [120]. In addition, in clinical practice, cancer patients often do not receive a single type of immunotherapy, and it is almost always combined with* a* chemotherapy regimen. Therefore, it is necessary to evaluate the effect of different anticoagulant drugs on the antitumor efficacy of complex immunotherapy/chemotherapy regimens. Conversely, when analyzing the effects of immunotherapy treatments on the risk of coagulation-related complications, variable incidence rates emerged of ICI-associated thrombosis: some studies indicate risks comparable to chemotherapy, while others report higher rates, especially in the early phases of treatment. In line with this, preclinical evidence supports that immune-checkpoint inhibitors (ICIs) effectively contribute to thrombotic events through multifactorial mechanisms, including disruption of immune homeostasis [121] and hyperactivation of T cells. This condition then leads to increased production of proinflammatory cytokines, such as TNF [122], induction of TF expression, and an increase in prothrombotic markers, including NETs and circulating nucleosomes. Additional mechanisms by which ICIs can promote thrombosis include impaired fibrinolysis [123] and dysregulation of myeloid-derived suppressor cells (MDSCs). The latter can increase vascular permeability and support abnormal angiogenesis, contributing to thrombosis risk. Moreover, MDSCs directly interact with platelets through checkpoint molecules, potentially inducing platelet activation or the release of TF–containing microparticles from monocytes [124]. Finally, beyond these indirect effects, ICIs can also directly interact with platelets and activate them [125].

### Clinical and non-clinical relevance of thromboembolic and hemorrhagic complications

Cancer-associated thromboembolic complications represent a clinical event with a relevant impact on survival and prognosis. Excluding disease progression, cancer-associated thrombosis is recognized as one of the leading causes of death in oncology patients. The occurrence of venous thromboembolism is associated with reduced overall survival (OS), higher rates of thrombotic recurrence, and a greater incidence of bleeding events compared with the non-oncologic population [126]. These complications negatively affect prognosis, even when they lead to interruption or reduction of anticoagulation in patients at high thrombotic risk [117]. Conversely, thromboembolic or hemorrhagic events can interfere with the continuity of anticancer therapies since they cause treatment delays, dose reductions, or temporary or permanent interruption of treatments, with potential negative effects on control of cancer progression [114]. Management of cancer-associated thrombosis is therefore closely intertwined with oncologic decision-making, as thrombotic events and their treatment can directly influence anticancer strategy and indirectly affect oncologic prognosis [95]. Coagulation-related complications also have a significant impact on healthcare systems and on patients’ lifestyle [112, 114]. From a healthcare system perspective, venous thromboembolism and major bleeding events often require hospitalization, prolonged length of stay, and additional diagnostic and therapeutic interventions, increasing care burden and resource use in terms of personnel, space, and time, both during active cancer treatment and in long-term risk management, with an overall increase in healthcare costs [112]. Regarding patient quality of life, pain, functional limitations, anxiety related to the risk of thrombotic recurrence or bleeding, and the need for prolonged anticoagulant therapy contribute to a perceived worsening of health status [114]. This impact is further amplified by the complexity and burden of treatment management for patients, including more visits and analysis, medications*,* and monitoring. The recommendations from international guidelines require adaptation of therapeutic strategies to different risk profiles, considering tumor type and site, thrombotic and bleeding risk, renal and hepatic function, thrombocytopenia, and disease course. Management of thrombosis in cancer patients requires a multidisciplinary approach and periodic reassessment of therapeutic choices. Application of these recommendations aims to improve safety and clinical outcomes by reducing thrombotic and hemorrhagic events, limiting unnecessary treatment interruptions, and optimizing *the* use of healthcare resources [114–116].

## Conclusions

Hemostasis can be considered a dynamic and context-dependent biological complex system. In cancer, this system is progressively reprogrammed at multiple spatial levels, from the primary TME to the systemic circulation, shaping local immune regulation, vascular remodeling, and the efficiency of metastatic dissemination. Tumor-associated activation of tissue factor signaling, platelet function, fibrin deposition, and immune cell crosstalk supports angiogenesis, immune evasion, and tumor cell survival. In this context, the relationship between cancer and coagulation factor cannot be viewed solely as a clinical complication, but rather as a manifestation of a broader biological convergence between malignancy, inflammation, and hemostatic control. Anticancer therapies further interact with these pathways by altering endothelial, platelet, and coagulation functions, thereby modulating both thrombotic risk and treatment tolerance. This interaction with coagulation, immune regulation, and tumor progression highlights the potential value of hemostatic biomarkers as indicators of disease aggressiveness and metastatic potential. At the same time, it would be useful to intervene on these biomarkers not only as molecules useful for prognosis/diagnosis but also as therapeutic targets to prevent thromboembolic events. Future work will require integrated molecular and clinical approaches to define patient-specific risk profiles and to determine how modulation of hemostatic pathways can be safely incorporated into personalized cancer treatment.

## Data Availability

Not applicable.

## Abbreviations

APTT: Activated Partial Thromboplastin Time
CAFs: Cancer-associated fibroblasts
CTCs: Circulating Tumor Cells
CYP3A4: Cytochrome3A4
DDIs: Drug–drug interactions
DOACs: Direct oral anticoagulants
eNOS: Endothelial Nitric Oxide Synthase
EVs: Extracellular Vesicles
FDP: Fibrin Degradation Products
F I: Fibrinogen
F II: Prothrombin
F IIa: Thrombin: Active form of prothrombin
F V: ProACCELERIN
F VII: Proconvertin
F VIII: Antihemophilic Factor A
F IX: Christmas Factor
F X: Stuart–Prower Factor
F XI: Plasma Thromboplastin Antecedent
F XII: Hageman Factor
F XIII: Fibrin-Stabilizing Factor: Cross-links fibrin
F Xa: Activated Factor X
GGCX: Gamma-glutamyl carboxylase
HIF-1α: Hypoxia-inducible factor-1α
HMWK: High Molecular Weight Kininogen
ICI: Immune Checkpoint Inhibitor
Mac–1: Integrin αMβ2
MDSCs: Myeloid-Derived Suppressor Cells
MHC-I: Major Histocompatibility Complex Class I
M2: Macrophages 2
NETs: Neutrophil Extracellular Traps
NK: Natural Killer
OS: Overall survivals
PAI–1: Plasminogen Activator Inhibitor–1
PAR: Protease-Activated Receptors
PDGF: Platelet-Derived Growth Factor
P-gp: P-glycoprotein
PT: Prothrombin Time
TAT: Thrombin-Antithrombin Complexes
TCIPA: Tumor Cell-Induced Platelet Aggregation
TE: Tumors Induce Tissue Endothelial
TEPs: Tumor-Educated Platelets
TF: Tissue Factor or Factor III
TGF-β: Transforming Growth Factor-β
TGF-β1: Transforming Growth Factor-β1
TKIs: Thyrosin kinase inhibitors
TME: Tumor Microenvironment
TNBC: Triple-Negative Breast Cancer
VEGF: Vascular Endothelial Growth Factor
VTE: Venous Thromboembolism
vWF: Von Willebrand Factor
